# Actinobacteria Community and Their Antibacterial and Cytotoxic Activity on the Weizhou and Xieyang Volcanic Islands in the Beibu Gulf of China

**DOI:** 10.3389/fmicb.2022.911408

**Published:** 2022-07-12

**Authors:** Lin Wang, Chunyan Peng, Bin Gong, Zicong Yang, Jingjing Song, Lu Li, Lili Xu, Tao Yue, Xiaolin Wang, Mengping Yang, Huimin Xu, Xiong Liu

**Affiliations:** ^1^The Guangxi Key Laboratory of Beibu Gulf Marine Biodiversity Conservation, College of Marine Sciences, Beibu Gulf University, Qinzhou, China; ^2^Guangxi Key Laboratory of Marine Disaster in the Beibu Gulf, Beibu Gulf University, Qinzhou, China; ^3^Sea Area Use Dynamic Supervising and Managing Center of Fangchenggang City, Fangchenggang, China

**Keywords:** Actinobacteria, volcanic island, microbiome, high-throughput sequencing, Beibu Gulf

## Abstract

Weizhou Island and Xieyang Island are two large and young volcanic sea islands in the northern part of the South China Sea. In this study, high-throughput sequencing (HTS) of 16S rRNA genes was used to explore the diversity of Actinobacteria in the Weizhou and Xieyang Islands. Moreover, a traditional culture-dependent method was utilized to isolate Actinobacteria, and their antibacterial and cytotoxic activities were detected. The alpha diversity indices (ACE metric) of the overall bacterial communities for the larger island (Weizhou) were higher than those for the smaller island (Xieyang). A beta diversity analysis showed a more dispersive pattern of overall bacterial and actinobacterial communities on a larger island (Weizhou). At the order level, Frankiales, Propionibacteriales, Streptomycetales, Micrococcales, Pseudonocardiales, Micromonosporales, Glycomycetales, Corynebacteriales, and Streptosporangiales were the predominant Actinobacteria. A total of 22.7% of the OTUs shared 88%–95% similarity with some known groups. More interestingly, 15 OTUs formed a distinct and most predominant clade, and shared identities of less than 95% with any known families. This is the first report about this unknown group and their 16S rRNA sequences obtained from volcanic soils. A total of 268 actinobacterial strains were isolated by the culture-dependent method. Among them, 55 *Streptomyces* species were isolated, representing that 76.6% of the total*. S. variabilis* and *S. flavogriseus* were the most abundant. Moreover, some rare Actinobacteria were isolated. These included *Micromonospora* spp., *Nocardia* spp., *Amycolatopsis* spp., *Tsukamurella* spp., *Mycobacterium* spp., and *Nonomuraea* spp. Among them, eight *Streptomyces* spp. exhibited antibacterial activity against *Bacillus cereus*. Only three strains inhibited the growth of *Escherichia coli*. Four strains showed good activity against aquatic pathogenic bacterial strains of *Streptococcus iniae*. The cytotoxicity assay results showed that 27 strains (10.07%) exhibited cytotoxic activity against HeLa and A549 cell lines. Many actinobacterial strains with cytotoxic activity were identified as rare Actinobacteria, which illustrated that volcanic islands are vast reservoirs for Actinobacteria with promising antibacterial and cytotoxic activity. This study may significantly improve our understanding of actinobacterial communities on volcanic islands. The isolated Actinobacteria showed promising prospects for future use.

## Introduction

Actinobacteria is an important phylum whose members have extensive applications. Actinobacteria are Gram-positive bacteria with higher G + C contents in their genomic DNA (Yadav et al., 2018; van Bergeijk et al., 2020). Actinobacteria can produce various secondary metabolites with medicinal properties, such as antibacterial (Assad et al., 2021; Majidzadeh et al., 2021), antiviral (Kim et al., 2016; Chen et al., 2018; Real et al., 2018), antifungal (Marasabessy et al., 2017; Ruangwong et al., 2022), and anticancer (Azman et al., 2017; Hozzein et al., 2021) properties. Moreover, these microbes also play an essential role in ecosystems. Actinobacteria contribute significantly to the process of nitrogen fixation (Nouioui et al., 2017), decomposition of organic matter such as cellulose and lignin (Javed et al., 2021), plant growth (Mitra et al., 2022), production of organic acids (Lasudee et al., 2021), and phosphate solubilization (Soumare et al., 2021) in soil. However, due to increasing difficulty in obtaining novel Actinobacteria from conventional environments, some special and untapped environments have attracted our attention (Hassan et al., 2019).

Weizhou Island and Xieyang Island are two large volcanic sea islands, and Weizhou Island is the youngest and largest volcanic sea island in the northern part of the South China Sea (Deng et al., 2017). They are typical insular systems that are valuable for studying microbial biogeographic patterns on islands. Weizhou Island is 20 nautical miles south of the mainland, with an area of 24.74 km^2^. Xieyang Island is approximately nine nautical miles southeast of Weizhou Island, with an area of 1.89 km^2^. Some scientists have suggested that the eruption events of Weizhou Island and Xieyang Island occurred during the early middle Pleistocene (1.42–0.49 Ma)/late Pleistocene (36–33 ka; Fan et al., 2006). After that, the cooled magma turned into rocks, and the formation of soils gradually emerged from the weathering of rocks. The soil on the two islands is mainly laterite, a type of red soil that develops from rock decay (Lan et al., 1996). Microbial communities played a significant role during the formation of soils derived from volcanoes (Joergensen and Castillo, 2001; Herrera and Cockell, 2007). There are several reasons for us to explore the actinobacterial communities of soil on Weizhou and Xieyang Island. First, these two islands are located in tropical regions with relatively high temperatures (16.2°C–26.3°C; Zhang et al., 2021) and ultraviolet radiation, and there is a shortage of fresh water in the soil (Deng et al., 2017). Second, the volcanic eruptions and Lava flows make the volcanic islands unique for their high temperatures. In the early stage of development, these virgin lands are nutrient-poor and are referred to as “volcanic deserts” (Yoshitake et al., 2013). These factors make the soil of the volcanic ecosystem an unusual setting for Actinobacteria survival. Although the eruption of Weizhou and Xieyang volcanic Islands passed millions of years ago and plants colonization of the islands has significantly altered the microbial communities, some untapped groups of Actinobacteria are believed to remain in these young volcanic ecosystems. Therefore, studying the microbial communities on Weizhou Island and Xieyang Island is urgently needed.

In the present study, to explore and understand actinobacterial communities on the Weizhou and Xieyang volcanic islands, a high-throughput sequencing technique based on the Illumina platform was utilized. Moreover, a traditional culture-dependent method was utilized to isolate Actinobacteria, and their antibacterial and cytotoxic activities were detected. Our goals were to answer the following questions: (1) the diversity of actinobacterial communities on Weizhou and Xieyang volcanic Island; and (2) the antibacterial and cytotoxic activity of actinobacterial strains and their application potential in the pharmaceutical industry. This study may significantly improve our understanding of actinobacterial communities on volcanic islands and the promising prospects for their utilization in future.

## Materials and Methods

### Sample Collection

**Figure 1:**
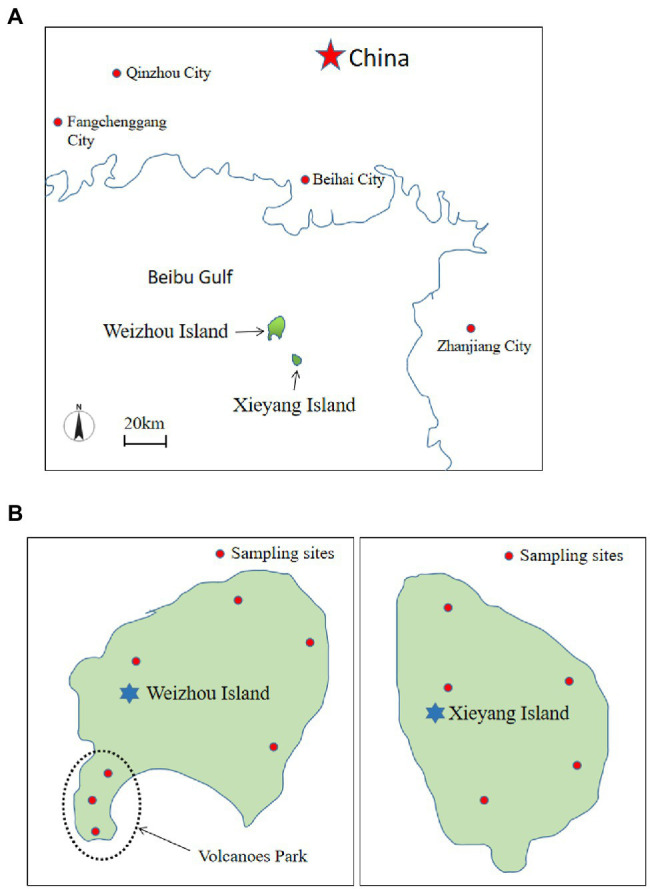
Geographical location of Weizhou and Xieyang Island **(A)** and sampling sites of both islands from which samples were collected **(B)**.

Samples were collected from Weizhou Island (20°54′-21°10’N, 109°00′-109°15′E) and Xieyang Island (20°89′-20°91’N, 109°20′-109°22’N; Figure 1A). Both islands have a tropical monsoon climate, with an annual average temperature of 22.6°C and average yearly precipitation of 138.02 cm (Zhang et al., 2021). The comparatively high accumulated temperature is suitable for weathering of the primary rock-forming minerals and the development of red weathering crust (Wilson, 2004). On Weizhou Island, there is a great variety of shrubs and trees. However, the vegetation on Xieyang Island is mainly grass (Su et al., 2017). Soil samples were collected at a depth of 10–20 cm using sterile soil samplers (three replicates were collected from a circular area measuring 1 m in diameter and then mixed), and seven samples were collected from Weizhou Island and five from Xieyang Island. The sampling regions of both islands from which samples were collected are indicated (Figure 1B). The soils collected from the sampling sites are primarily laterite, a soil developed from volcanic rock, without any farming activity. Human activities seldom affected any of the sampling sites. All the samples were collected in sterile plastic bags and were immediately transferred to the laboratory. Each soil sample was homogenized and divided into three parts: one part was used for chemical composition analysis; the second part was used to isolate Actinobacteria; and the rest of the soils were stored at −80°C until DNA extraction. The chemical composition [NO_3_^−^-N, NH_4_^+^-N, total carbon contents (TC), total nitrogen elements (TN), total sulfur elements (TS), Fe, and total phosphorus elements (TP)] of the soils were measured and are described in Supplementary Figure S1.

### Total DNA Extraction, Illumina MiSeq Sequencing

The total genomic DNA of soils was extracted with a DN45 Fast Beat Soil DNA extraction kit (Aidlab Biotechnologies Co., Ltd) following the manufacturer’s protocols. The obtained DNA was used for PCR amplification. The V3-V4 region of the 16S rRNA gene was amplified with the primers (Forward: CTACGGGNGGCWGCAG; Reverse: GACTACHVGGGTATCTAATCC) described previously (Wu et al., 2016). PCR amplifications were conducted using Platinum SuperFi II DNA Polymerase (Thermo Fisher Scientific Inc.). The process was carried out according to the procedure provided by Sun et al. (2018). The products were analyzed and purified (Gong et al., 2019a), sequencing libraries were generated, and library quality was assessed according to a previous article (Gong et al., 2019b). Finally, the purified amplicons were sequenced using HiSeq PE250. After the raw data sequences were generated, primers and barcode sequences were removed, and FLASH was utilized for merging the paired-end reads (Magoč and Salzberg, 2011). QIIME 2 software (V1.7.0) was used to filter the raw tags under our filtering conditions (the length of the continuous high-quality base was less than 75% of the tag length), and then the chimera and singleton sequences were removed to obtain high-quality clean tags (Bolyen et al., 2019). The OTUs were classified according to a previously described method (Wang et al., 2007). For each OTU, a representative sequence was selected, and the SILVA Database (SSU 132) was used to assess the taxonomic affiliation (Quast et al., 2012). The above sequencing and bioinformatics analysis were conducted by a commercial company, Majorbio (Shanghai, China). All the sequence data were uploaded to the National Center for Biotechnology Information (NCBI) Sequence Read Archive (SRA) under the BioProject number PRJNA684667.

### Bioinformatics Analysis of 16S rRNA Gene Amplicon Data

The 20 most abundant genera within Actinobacteria were identified. Representative sequences were extracted from sequence database files using TBtools (Chen et al., 2020) and then blasted in GenBank^1^ for the closest related sequences (McGinnis and Madden, 2004). The most closely related sequences of the Actinobacteria type strains were selected. All sequences mentioned above were retrieved and aligned using CLUSTALW 2.1 (Thompson et al., 1994). The neighbor-joining (NJ) method was employed to construct a phylogenetic tree using MEGA 5.0 (Tamura et al., 2011). Bootstrap values were calculated for 1,000 replications.

The ACE, Chao1, Shannon, and Simpson diversity indices were calculated to indicate the alpha diversity (Wagner et al., 2018). PCA was used to evaluate differences in bacterial community complexity among samples (Alizadeh Behbahani et al., 2017). An analysis of similarities (ANOSIM) test was used to calculate whether the above differences were statistically significant (Somerfield et al., 2021). LDA effect size (LEfSe) analysis was employed to determine different bacterial and archaeal taxa between the Weizhou and Xieyang Islands (Takezawa et al., 2021). The relationship between microbial functional groups and soil nutritional elements (NO_3_^−^-N, NH_4_^+^-N, TN, TC, TS, Fe, TP) was analyzed by Redundancy analysis (RDA) with vegan package in R (Legendre and Anderson, 1999).

### Statistical Analysis

SAS software (v9.4) was used to conduct statistical analyses, and a 95% confidence level was employed to determine whether the differences were significant.

### Isolation and 16S RNA Gene Identification of Actinobacteria

Three to 5 g of soil from each sampling site (Figure 1) was ground into powder and added to 20 ml of sterile physiological saline (0.9%), and then incubated at 28°C ± 2°C, and 190 rpm in shaker incubator for 30–60 min. The soil suspension was serially diluted. Two hundred microliters of the dilutions (10^−2^, 10^−3^, and 10^−4^) were placed onto each of the media and incubated at 28°C for 7–28 days. Actinobacteria were isolated using the following media:

GS: soluble starch 20 g, KNO_3_ 1 g, K_2_HPO_4_·3H_2_O 0.5 g, MgSO_4_·7H_2_O 0.5 g, FeSO_4_·7H_2_O 0.1 g, agar 15 g, and pure water 1 l (Chen et al., 2019a).

HSG: humic acid 0.1 g, CaCl_2_ 0.02 g, FeSO_4_·7H_2_O 1 mg, MnCl_2_·4H_2_O 1 mg, ZnSO_4_·7H_2_O 1 mg, NiSO_4_·6H_2_O 1 mg, CHES 5 mmol, gellan gum 7 g, agar 15 g, and pure water 1 l (Li et al., 2021a).

HV: humic acid 1 g, CaCO_3_ 0.02 g, Na_2_HPO_4_ 0.5 g, KCI 0.5 g, MgSO_4_·7H_2_O 0.5 g, FeSO_4_·7H_2_O 0.1 g, agar 15 g, and pure water 1 l (Li et al., 2021a).

ATCC: glucose 1 g, soluble starch 2 g, malt extract 0.5 g, CaCO_3_ 0.05 g, N-Z-Amine 0.5 g, agar 15 g, and pure water 1 l (produced by a commercial company, Hope Bio, in Qindao, China).

All the media were adjusted to a pH of 7.2–7.4. Additionally, naphthylpyruvic acid (100 μg/ml), kanamycin (50 μg/ml), ampicillin (100 μg/ml), or 3% potassium dichromate (1 ml) were added to inhibit microbial growth. Actinobacterial colonies were picked with sterile toothpicks and re-streaked onto another medium for purification. The purified cultures were stored in glycerol stock (20%–25%, v/v) at −80°C.

### Cultivation and Identification of Isolated Actinobacteria

The actinobacterial isolates were identified according to their characteristics. After the isolates were purified three times, they were cultivated in media comprised of 20 g/l soluble starch, 5 g/l glucose, 10 g/l malt extract, 0.5 g/l K_2_HPO_4_·3H_2_O, 0.3 g/l CaCO_3_, and 20 g/l NaCl (pH 7.2–7.4; Gong et al., 2018). After the Actinobacteria were cultivated under shaking conditions (200 rpm) for 7 days, the culture was divided into two parts. A 1.5 ml culture was maintained in glycerol suspensions (25%, v/v) at −70°C, and 3–5 ml culture was centrifuged for total genomic DNA extraction using a previously described method (Gong et al., 2018). The 16S rRNA genes were PCR amplified using primers 27f (5′-AGAGTTGATCCPATGGCTCAG-3′) and 1541R (5′-AAGGAGGTGATCCAGCC-3′) according to the procedure described by Gong et al. (2018). The PCR products were purified with a DNA Gel Extraction Kit (Sigma-Aldrich Co. LLC) and sequenced by a commercial company, GENEWIZ LLC, in China. The obtained sequences were compared with the EzBioCloud database.^2^

### Detection of Antibacterial and Cytotoxic Activity

The antibacterial activity was investigated using the method described by Gong et al. (2018). *B. cereus* ATCC 14579, *E. coli* ATCC 13706 and *S. iniae* ATCC 29178 were used as indicator strains. We selected *B. cereus* and *E. coli* (representing Gram-positive and Gram-negative bacteria, respectively) as indicator strains, because they are widely used by other scientists for similar research. This selection will facilitate the comparison of our results with theirs. *S. iniae* is one of the most serious aquatic pathogens causing high losses in farmed marine and freshwater fish (Agnew and Barnest, 2007). Antimicrobial susceptibility was tested using the Kirby-Bauer disk diffusion method (Biemer, 1973). The fermentation broth was centrifuged, and the supernatant was diluted 100 times and then used for cytotoxic activity analysis. In the cytotoxic activity analysis, an MTT assay using the human lung adenocarcinoma cell line (A549) and human cervical cancer cell line (HeLa) as indicators was conducted, and the growth inhibition rate was calculated (Mosmann, 1983).

## Results

### Alpha and Beta Diversity of Samples

**Figure 2:**
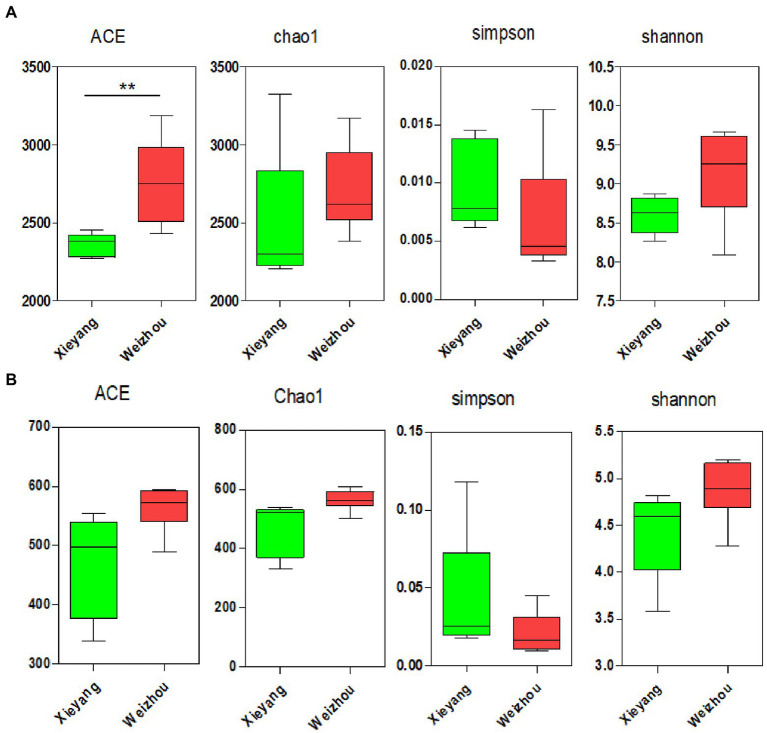
The alpha diversity of bacterial and actinobacterial communities in Weizhou and Xieyang Islands is illustrated by ACE, Chao1, Shannon’s, and Simpson’s diversity indices. **(A)** The alpha diversity of the overall bacterial communities; **(B)** the alpha diversity of the actinobacterial communities on Weizhou and Xieyang Islands. Asterisks indicate the significance of the ANOVA results (^**^p < 0.01).

A total of 678,025 good quality (clean) reads were recovered from the samples, and the average read was 56,502 for each soil sample. After quality filtering, they were clustered into 6,447 OTUs. The observed species ranged from 1,985 to 7,423, with an average of 3,687. The ACE metric of Weizhou Island was significantly higher than that of Xieyang Island (*t*-test, *p* < 0.01). However, the Chao1, Shannon’s, and Simpson’s diversity indices showed no apparent differences among them (*t*-test, *p* > 0.05; Figure 2A). Moreover, the alpha diversity indices of the actinobacterial community of Weizhou and Xieyang Islands showed no significant differences (*t*-test, *p* > 0.05; Figure 2B).

**Figure 3:**
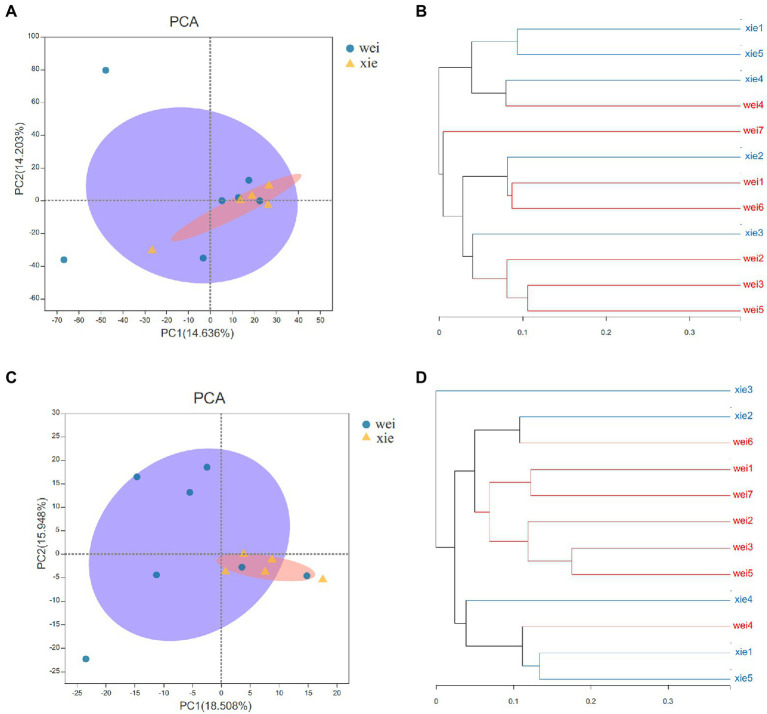
Principal component analysis (PCA) plots and UPGMA tree based on the weighted UniFrac distance metric of microbial and actinobacterial communities from the Xieyang and Weizhou Islands using 16S rRNA gene amplicon data. **(A)** PCA plots of the entire bacterial community profiles. The microbial communities from Xieyang Island formed a closer group than those from Weizhou Island (*t*-test, *p* < 0.05; two-sample Wilcoxon test, *p* < 0.01). **(B)** The UPGMA tree of the whole bacterial communities. No significant differences were indicated between the Weizhou and Xieyang Islands (Anosim, R = 0.105, *p* > 0.05). **(C)** PCA plots of actinobacterial community profiles. The actinobacterial community from Xieyang Island formed a closer group than those from Weizhou Island (*t*test, *p* < 0.05; two-sample Wilcoxon test, *p* < 0.01). **(D)** The UPGMA tree of the actinobacterial community. No significant differences are shown in the actinobacterial community between the Weizhou and Xieyang Islands (Anosim, R = 0.279, *p* > 0.05) were shown. All the data were normalized before being subjected to statistical analysis.

**Figure 4:**
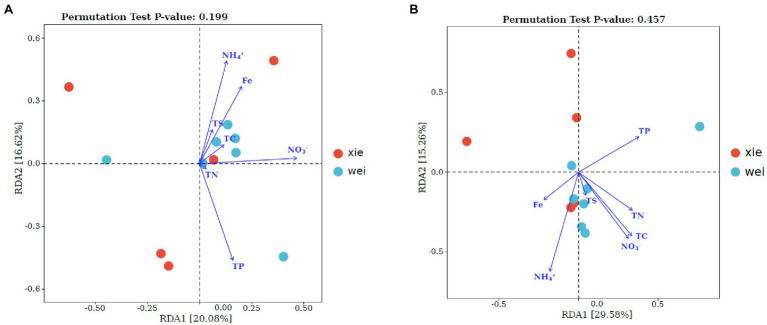
A redundancy analysis (RDA) was used to analyze the impact of the soil nutritional elements (NO_3_^−^-N, NH_4_^+^-N, TN, TC, TS, Fe, and TP) on the bacterial and actinobacterial communities. The blue arrows indicate the correlation between the nutritional elements and the bacterial **(A)** or actinobacterial **(B)** community structure. A Monte Carlo permutation test was used to evaluate the significance of these correlations.

The weighted UniFrac distance metric showed that the overall microbiota and actinobacterial community from Xieyang Island formed a closer group than those from Weizhou Island (*t*test, *p* < 0.05; two-sample Wilcoxon test, *p* < 0.01). This result was clearly illustrated by principal component analysis (PCA) plots (Figures 3A,C). Moreover, the UPGMA tree showed that there were no significant differences in the overall bacterial populations between the Weizhou and Xieyang Islands (Anosim, R = 0.105, *p* > 0.05; Figure 3B). A similar result was illustrated in the actinobacterial community between these two islands (Anosim, R = 0.279, *p* > 0.05; Figure 3D). A redundancy analysis (RDA) was used to analyze the impact of the soil nutritional elements (NO_3_^−^-N, NH_4_^+^-N, TN, TC, TS, Fe, and TP) on the bacterial (Figure 4A) and actinobacterial (Figure 4B) community. However, no significantly correlations of soil nutritional elements between Weizhou and Xieyang Island were illustrated (Monte Carlo permutation test, *p* > 0.05; Figure 4).

**Figure 5:**
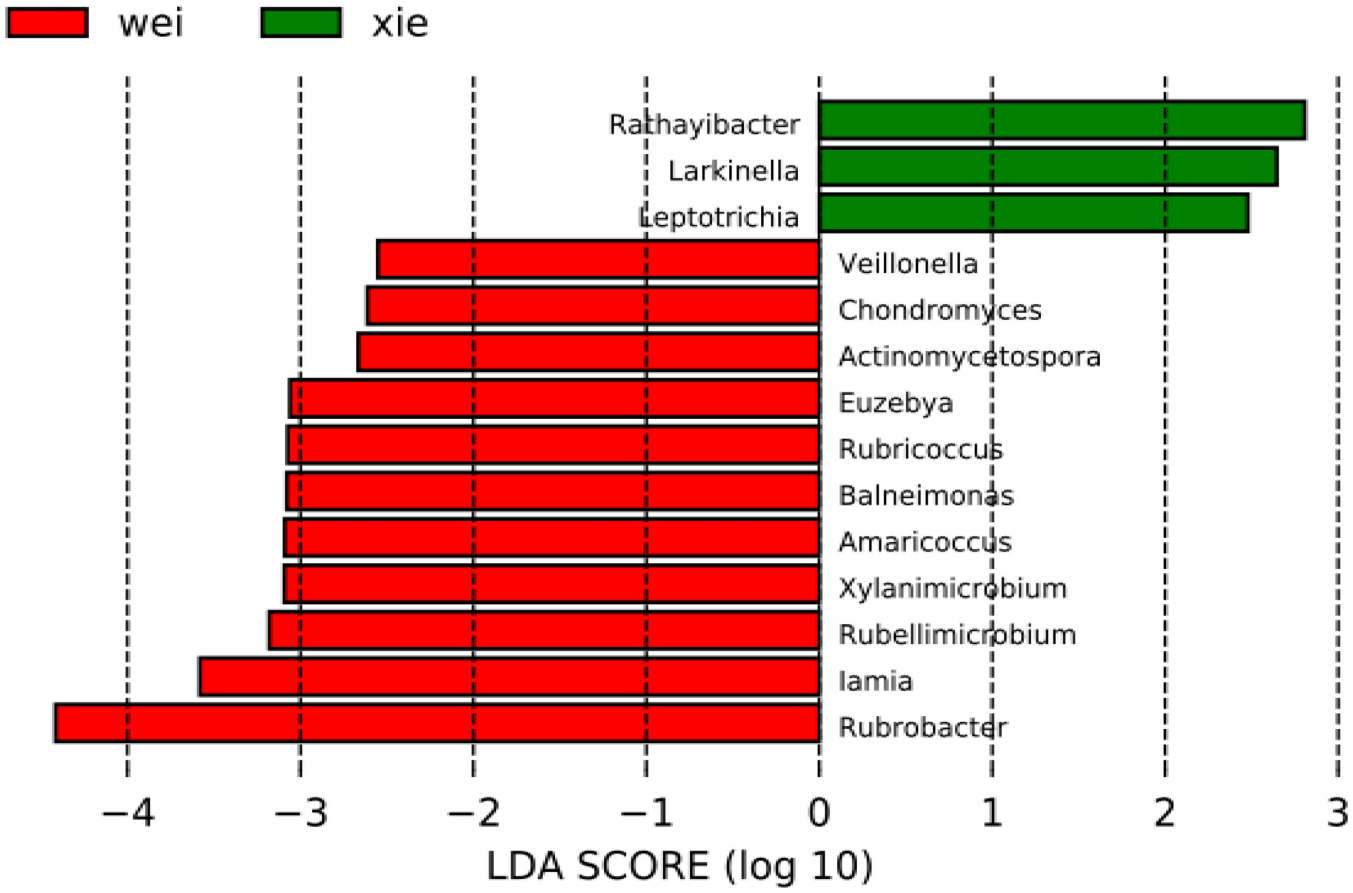
Linear discriminant analysis (LDA) and effect size (LEfSe) assay of microbial communities of the Weizhou and Xieyang Islands. The size of differentiation between Weizhou and Xieyang Islands was identified using LDA score with a threshold value of 2.5. “wei” = soil samples from Weizhou Islands. “xie” = soil samples from Xieyang Islands.

LEfSe analyses indicated that the abundance of several genera differed between the Wei and Xie groups (Figure 5). The relative abundance of the genera *Rathayibacter*, *Larkinella*, and *Leptotrichia* was higher on Xieyang Island. In comparison, the genera *Veillonella*, *Chondromyces*, *Actinomycetospora*, *Euzebya*, *Rubricoccus*, *Balneimonas*, *Amaricoccus*, *Xylanimicrobium*, *Rubellimicrobium*, *Iamia*, and *Rubrobacter* were more abundant on Weizhou Island. Among these, *Rathayibacter*, *Actinomycetospora*, *Euzebya*, *Xylanimicrobium*, *Iamia*, and *Rubrobacter* were assigned to Actinobacteria (Figure 5).

**Figure 6:**
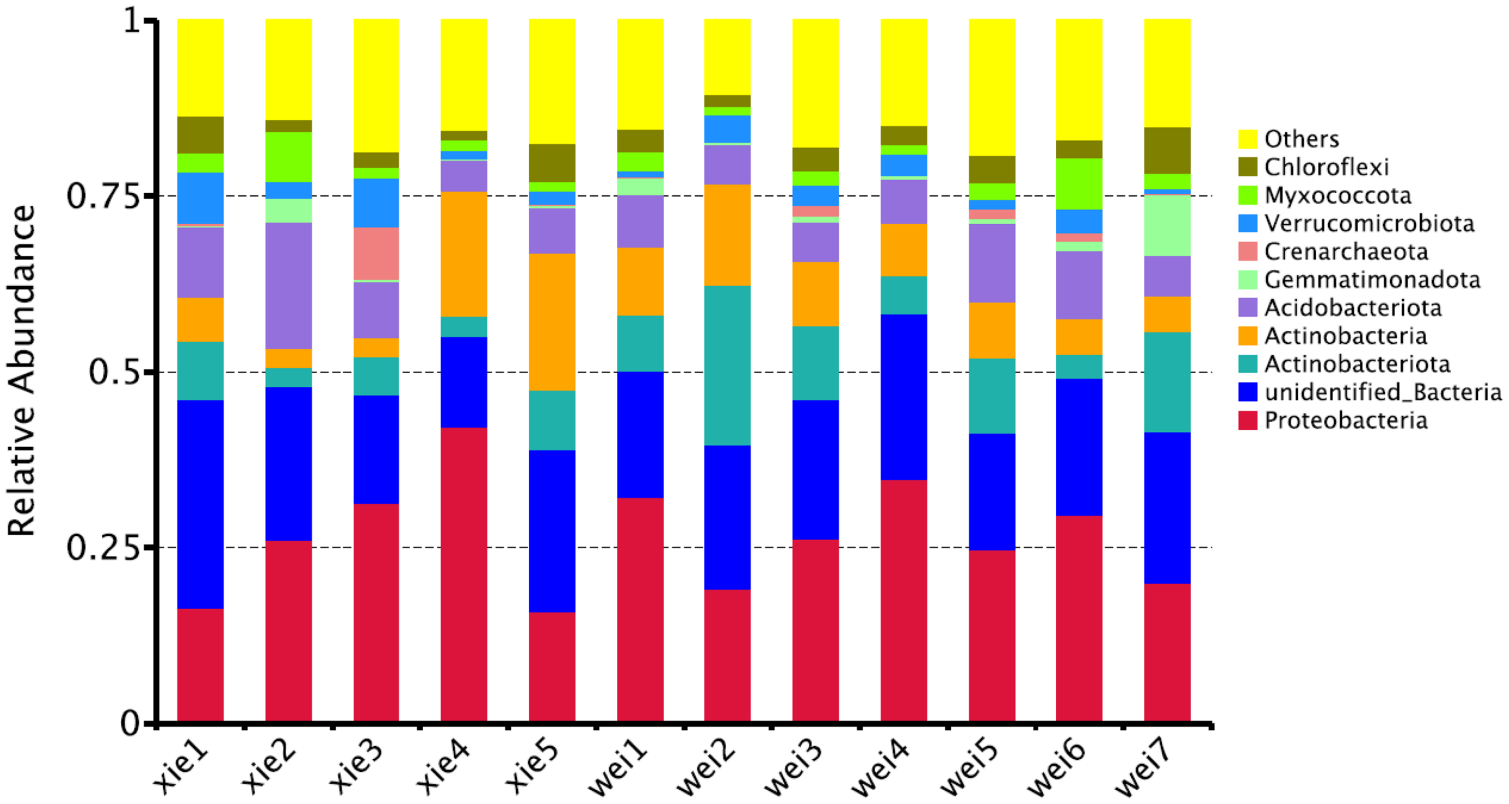
The relative abundance of the bacterial communities at the phylum taxon level by high-throughput sequencing (HTS)-based analysis. “xie1–xie5” and “wei1–wei7” indicate soil samples from Xieyang and the Weizhou Islands, respectively.

**Figure 7:**
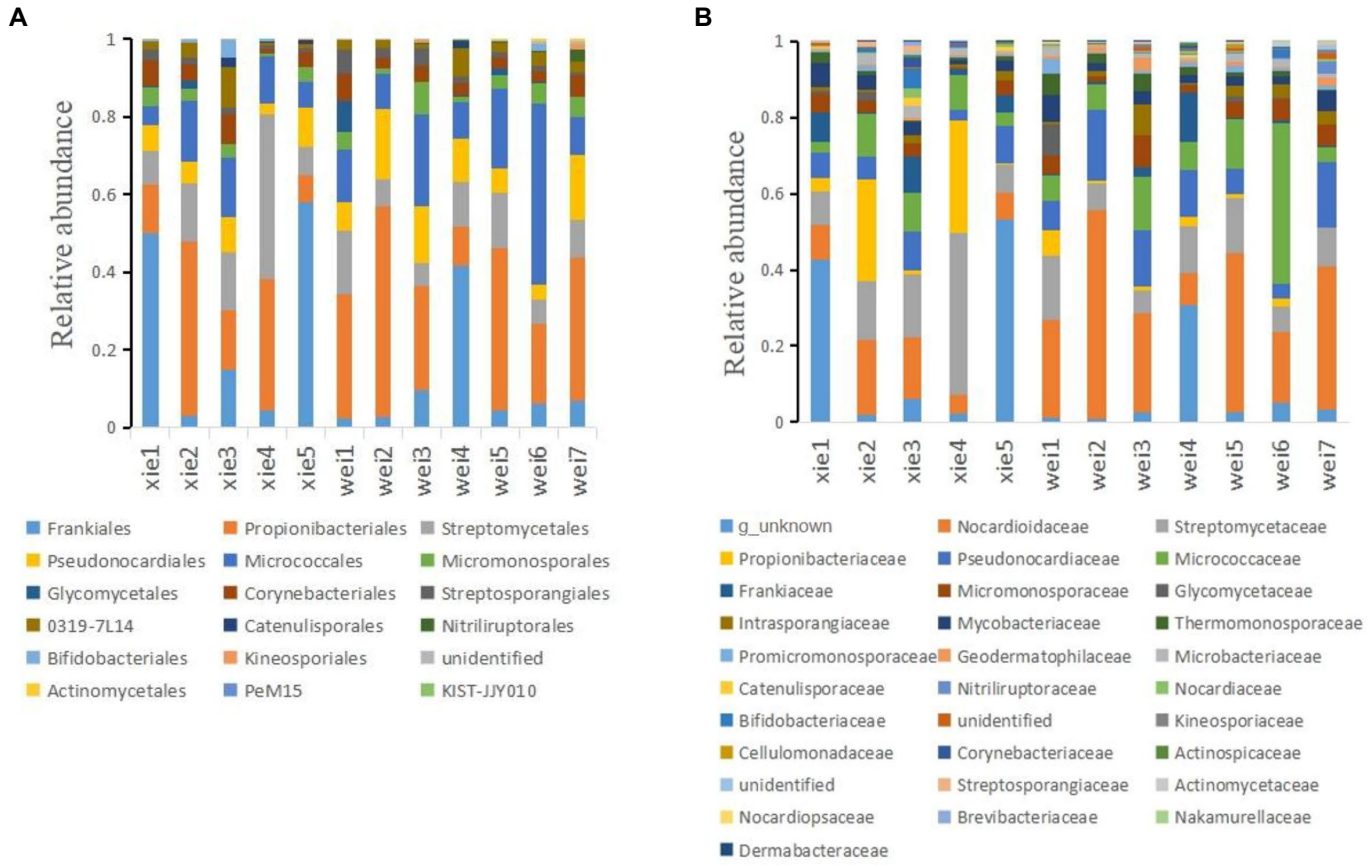
The relative abundances of different Actinobacteria at different taxon levels on the Weizhou and Xieyang Islands according to an HTS-based analysis. **(A)** Order taxon level; **(B)** family taxon level. “xie1–xie5” and “wei1–wei7” indicate soil samples from Xieyang and the Weizhou Islands, respectively.

**Figure 8:**
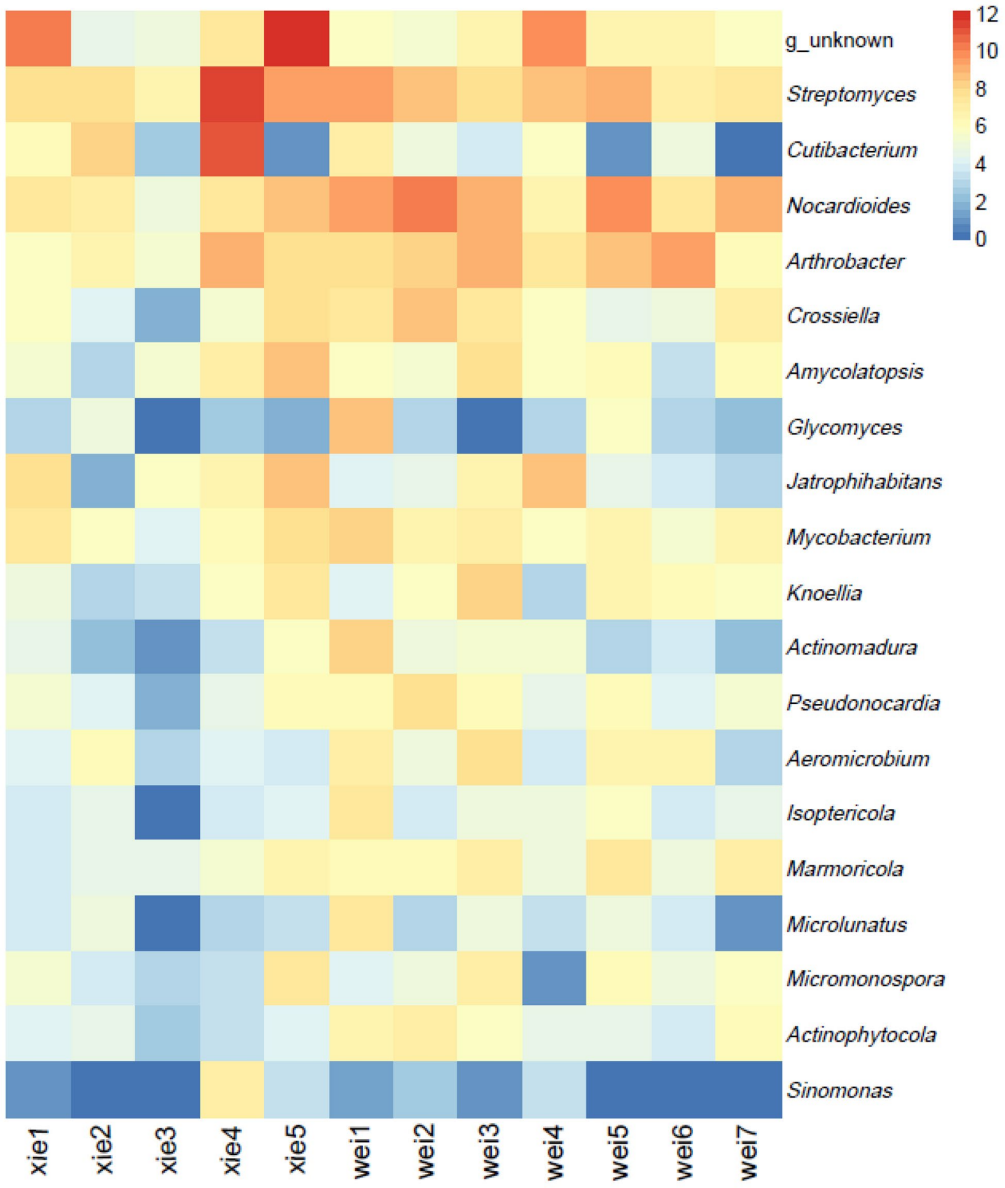
The most predominant genera of Actinobacteria on Weizhou and Xieyang Islands according to an HTS-based analysis. The “blue to red” color gradient indicates the relative abundance of the 20 most predominant genera of Actinobacteria. A deeper red indicates a higher relative abundance, while a deeper blue indicates a lower relative abundance. The values of 0, 2, 4, 6, 8, 10, and 12 indicate the value of relative abundance (the data were normalized and log2-transformed before analysis). “Xie1–Xie5” and “Wei1–Wei7” indicate soil samples from Xieyang and the Weizhou Islands, respectively.

The majority of OTUs could be assigned to 58 known phyla, while ~0.3% of OTUs could not be classified into any known phyla. According to the average relative abundance, Proteobacteria was the most predominant phylum (26.60%), followed by Actinobacteria (17.54%), Acidobacteria (8.18%), Gemmatimonadetes (1.58%), Crenarchaeota (1.06%), Verrucomicrobiota (2.98%), Myxococcota (2.76%), and Chloroflexi (3.33%; Figure 6). At the order level, Frankiales (1.77%), Propionibacteriales (2.48%), Streptomycetales (1.35%), Micrococcales (1.19%), Pseudonocardiales (0.85%), Micromonosporales (0.31%), Glycomycetales (0.08%), Corynebacteriales (0.33%), Streptosporangiales (0.19%), and 0319-7 L14 (0.2%) were the predominant actinobacterial populations across all the samples (Figure 7A). The most predominant families were Nocardioidaceae (1.88%), f_unknown (1.39%), Streptomycetaceae (1.35%), Pseudonocardiaceae (0.85%), Micrococcaceae (0.79%), Propionibacteriaceae (0.60%), and Frankiaceae (0.26%; Figure 7B). Lastly, the most predominant genera of Actinobacteria were g_unknown (1.38%), *Streptomyces*_ (1.35%), *Nocardioides* (1.02%), *Arthrobacter* (0.69%), *Cutibacterium*_ (0.54%), *Crossiella* (0.28%), and *Amycolatopsis* (0.23%; Figure 8).

**Figure 9:**
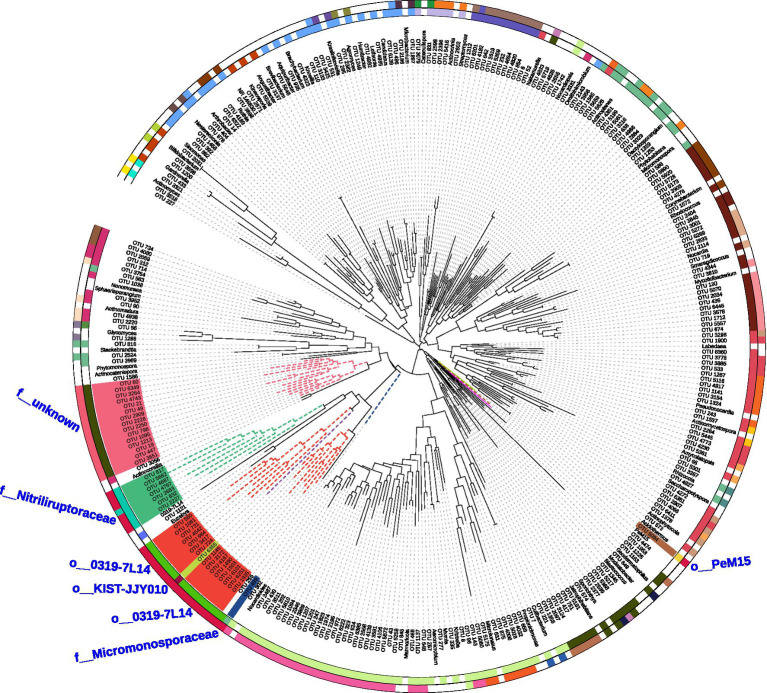
The phylogenetic tree of Actinobacteria OTUs. The branches of the phylogenetic tree labeled with different colors indicate that the genus formed a unique clade and shared <95% of its identity with some known families.

All 256 OTUs belonging to the Actinobacteria phylum were employed to construct a phylogenetic tree (Figure 9). From the tree, 77.3% of OTUs were found to formed clades into some known genera of Actinobacteria with a sequence similarity >97%. However, 16 OTUs, which were classified into order 0319-7 L14, shared identities of less than 91% of their identities with the known families. Fifteen OTUs formed a unique clade and shared identities of <95% with any known families. Six OTUs could be assigned to the family Nitriliruptoraceae (sequence similarity 90.71%–92.18%) but could not be classified into any known genera. OTU 499 could be classified into the family Micromonosporaceae with sequence similarities of 94.87%–95.35% with the nearest genus *Micromonospora*. OTU_5094 could be classified into order PeM15, but shared identities of less than 95% with the known families.

### Isolation of Actinobacterial Strains

**Figure 10:**
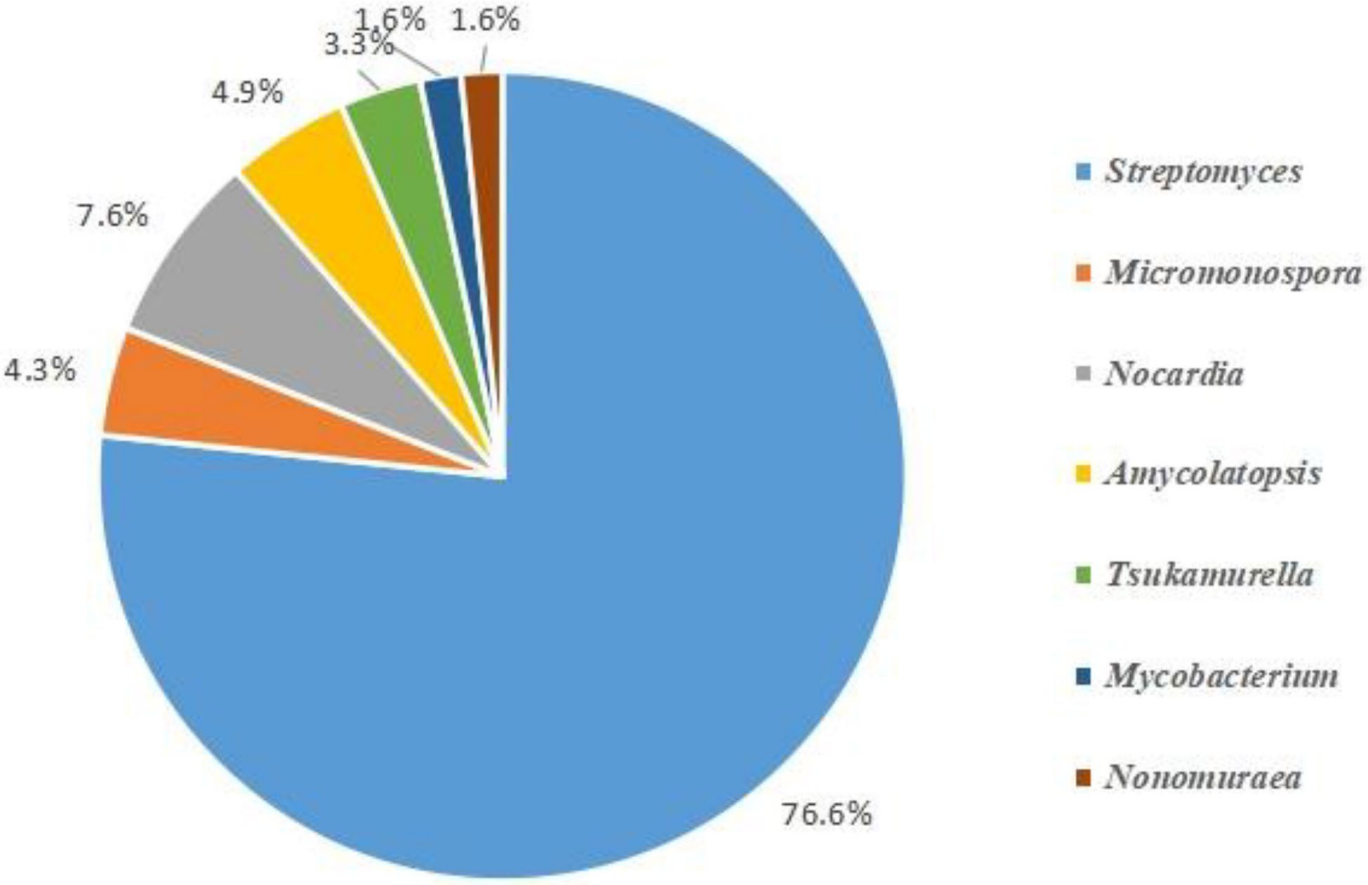
Different genera of actinobacterial strains isolated by traditional culture-dependent method.

**Figure 11:**
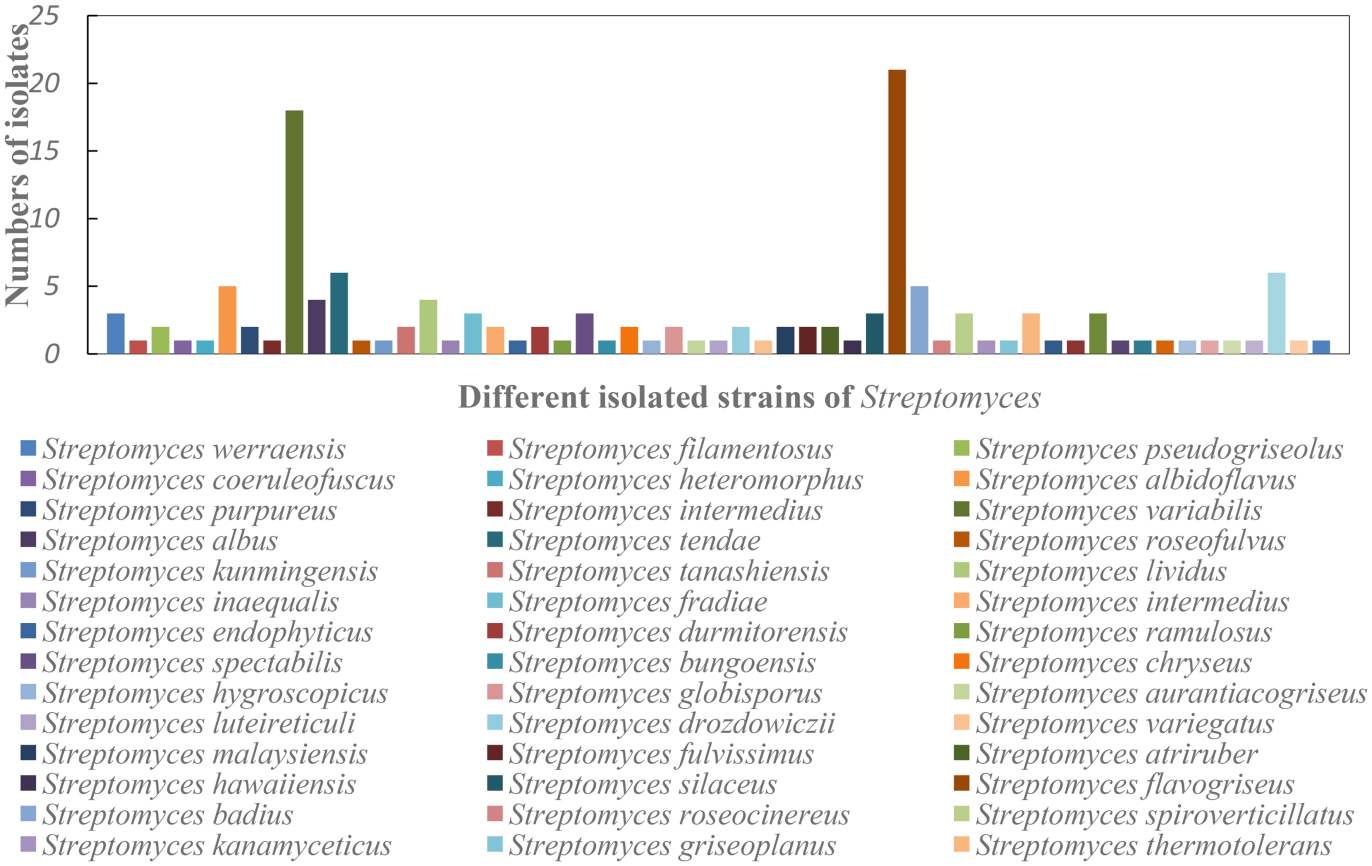
The number of isolates in each species of *Streptomyces*. The taxonomic classification of the isolates was predicted using the most related 16S rRNA gene.

**Figure 12:**
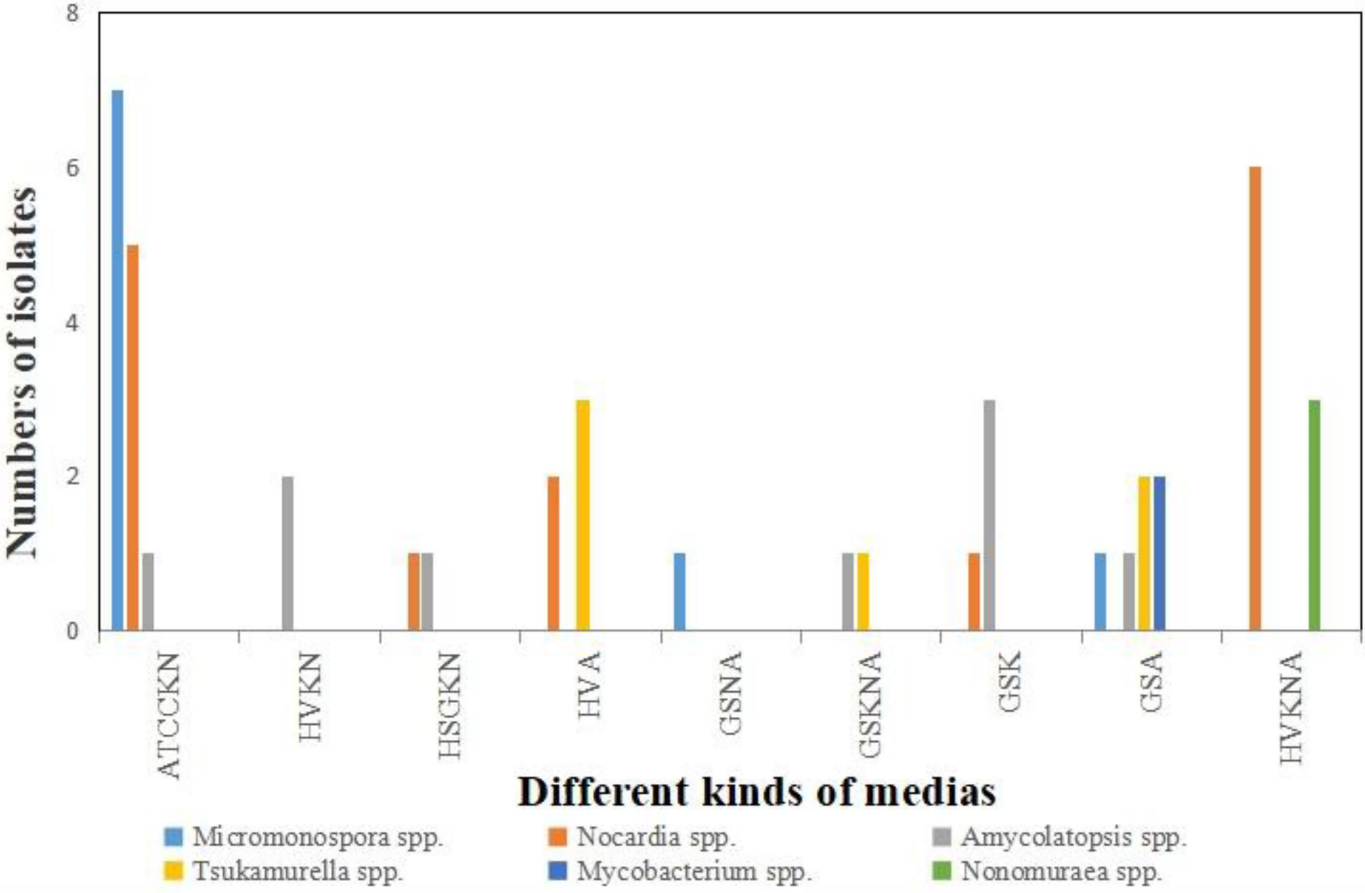
The number of rare Actinobacteria recovered from each selective medium.

A total of 268 actinobacterial strains were isolated from the Weizhou and Xieyang Islands. The 16S rRNA genes of 193 actinobacterial strains were sequenced. Among them, *Streptomyces* was the most predominant genus. A total of 141 isolates belonging to *Streptomyces* were isolated and purified, representing 76.6% of the total isolated Actinobacteria (Figure 10). These 141 strains were classified into 55 *Streptomyces* species. *Streptomyces variabilis* and *Streptomyces flavogriseus* were the most abundant species in the soil samples and comprised 12.8% and 15% of the isolated *Streptomyces* species, respectively (Figure 11). Moreover, some rare Actinobacteria were isolated and identified by 16S rRNA gene sequences. These included *Micromonospora* spp., *Nocardia* spp., *Amycolatopsis* spp., *Tsukamurella* spp., *Mycobacterium* spp., and *Nonomuraea* spp.; of these, *Micromonospora* spp., *Nocardia* spp., and *Amycolatopsis* spp. were the dominant groups. Based on the similarity of 16S rRNA gene sequences, five *Micromonospora* spp. most closely related to *M. echinospora* (four isolates), *M. aurantiaca* (one isolate), *M. chersina* (two isolates), *M. coxensis* (one isolate), and *M. fulviviridis* (one isolate) were obtained. Five *Nocardia* spp. were obtained from samples, and they were *N. crassostreae* (three isolates), *N. brasiliensis* (five isolates), *N. cyriacigeorgica* (one isolate), *N. asteroides* (five isolates), and *N. africana* (one isolate). Four *Amycolatopsis* species were isolated: *A. lurida* (one isolate), *A. vancoresmycina* (one isolate), *A. japonica* (two isolates), and *A. samaneae* (five isolates; Figure 10). The observed species of rare Actinobacteria isolated with ATCCKN media was the most abundant, comprising seven different species and three distinct genera, followed by GSA and HVA media and HVKNA media (Figure 12).

### Antibacterial and Cytotoxic Activity

All 268 actinobacterial strains were used for antibacterial and cytotoxic activity analysis (Table 1). Among them, eight actinobacterial strains (three most related to *S. variabilis,* three *Streptomyces fradiae,* one *Streptomyces albidoflavus,* and one *Streptomyces tanashiensis*) exhibited antibacterial activity against *B. cereus*. Only three actinobacterial strains inhibited the growth of the Gram-negative bacterium *E. coli*. Four strains (two most related to *S. variabilis,* one *S. fradiae,* and one *S. flavogriseus*) showed good activity against the aquatic pathogenic bacterium *S. iniae*. The cytotoxicity assay results showed that 27 strains (10.07%) exhibited cytotoxic activity against the HeLa and A549 cell lines.

**Table 1:** Antibacterial and cytotoxic activity of actinobacterial strains isolated from soils of the Weizhou and Xieyang Islands.

Strain no.	Putative species based on 16S rRNA gene sequences	Antibacterial activity^a^			Cytotoxic actions^b^	
		*B. cereus*	*E. coli*	*S. iniae*	HeLa	A549
HVAJ-2	*Streptomyces variabilis*	−	−	−	++	++
HVAJ-3	*Streptomyces albidoflavus*	+	−	−	++	++
HVAJ-5	*S. variabilis*	−	−	−	++	++
HVAJ-7	*S. variabilis*	−	−	−	++	++
HVAJ-8	*S. variabilis*	−	−	++	++	++
HVAJ-11	*S. variabilis*	+++	+	−	−	−
HVAJ-12	*S. variabilis*	+++	−	++	++	++
HVAJ-21	*S. variabilis*	−	−	−	++	++
HVAB-5	*Streptomyces fradiae*	+++	−	−	+	+
HVAB-6	*S. variabilis*	++	+	−	−	−
HVAC-6	*Tsukamurella tyrosinosolvens*	−	−	−	++	++
HVAG-11	*Streptomyces flavogriseus*	−	−	++	++	++
HVAG-16	*S. flavogriseus*	−	−	−	++	++
HVAG-19	*S. flavogriseus*	−	−	−	++	++
HVKNaJ-1	*Nocardia vulneris*	−	−	−	++	++
HVKNaG-3	*Nonomuraea jabiensis*	−	−	−	++	++
HVKNaG-5	*Nonomuraea jabiensis*	−	−	−	++	++
HVKNaG-8	*Streptomyces malaysiensis*	−	−	−	++	++
HVKNaH-7	*Nocardia crassostreae*	−	−	−	++	++
GSAD-1	*Mycobacteroides saopaulense*	−	−	−	++	++
GSAD-3	*Streptomyces drozdowiczii*	−	−	−	++	++
GSAE-6	*Streptomyces pseudovenezuelae*	−	−	−	++	++
GSAE-9	*Streptomyces albireticuli*	−	−	−	++	++
GSAF-2	*S. flavogriseus*	−	−	−	++	++
GSAG-4	*Mycobacteroides abscessus*	−	−	−	++	++
GSAI-5	*S. albireticuli*	−	−	−	++	++
GSKC-2	*Amycolatopsis samaneae*	−	−	−	++	++
GSAJ-2	*S. albireticuli*	−	−	−	++	++
GSAJ-4	*Streptomyces tanashiensis*	+++	+	−	−	−
GSKNaG-2	*T. tyrosinosolvens*	−	−	−	++	++
ATCCKNF11	*S. fradiae*	+++	−	+	−	−
ATCCKNF26	*S. fradiae*	+++	−	−	−	−

a Antibacterial activity illustrated by diameter of inhibition zone, “+” means 8.0 mm < inhibition zone < 10.0 mm, “++” means 10.0 mm < inhibition zone < 20.0 mm, “+++” means inhibition zone > 20.0 mm, “−” means antibacterial activity not been detected.

b Cytotoxic actions illustrated by growth inhibition rate of HeLa and A549, “+” means 50% < growth inhibition rate < 70%, “++” means growth inhibition rate > 70%, “−” means cytotoxic activity not been detected.

## Discussion

Actinobacteria are valuable for producing an extensive spectrum of secondary metabolites (Jose et al., 2021). The Weizhou and Xieyang volcanic islands formed ~3,000 to 1,000,000 years ago, and Weizhou Island is the largest and youngest volcanic island in China (Fan et al., 2006; Liao et al., 2019). These islands are located in tropical regions and are unique for their relatively higher temperatures, ultraviolet radiation, and shortage of fresh water (Deng et al., 2017). Recently, although the eruption of Weizhou and Xieyang volcanic islands has occurred for millions of years, we found that some thermophilic/thermotolerant actinobacteria, such as g__Acidothermus and f__Thermomonosporaceae, still remain in these volcanic ecosystems. Moreover, the actinobacterial communities of the two islands may have independent and unique phylogenetic signatures for geographical isolation. Although some soils have been reclaimed and affected by anthropogenic disturbances, many areas have seldom been affected by human activities (Chen et al., 2019b). The study of actinobacterial communities in these island soils may be significant for improving our understanding of Actinobacteria in these habitats, which are special and untapped among all those on Earth.

It should be mentioned that some alpha diversity indices for Weizhou are higher than those for Xieyang. Moreover, based on a beta diversity analysis, a more dispersive pattern of overall bacterial and actinobacterial communities was detected on Weizhou Island. Notably, the area of Weizhou was larger, and the plant diversity and species richness in Weizhou were higher than those in Xieyang. Compared to the primary colonization by grass (primarily *Acacia confusa* and *Opuntia dillenii*) on Xieyang Island, the vegetation on Weizhou Island is more diverse and is composed of 131 vascular species belonging to 121 genera and 74 families (Su et al., 2017). Moreover, three characteristics of Weizhou and Xieyang Islands should be considered: (1) they are geographically isolated; (2) they are not significantly affected by anthropogenic disturbance; and (3) the soil physicochemical factors of the two islands are similar (Ge and Han, 2009). During the early stage, the volcanic soils were nutrient-poor ecosystems and were referred to as “volcanic deserts” (Yoshitake et al., 2013). How did the microbiota form and develop from these “volcanic deserts”? Yoshitake et al. noted that microbial succession in the volcanic desert was significantly affected by colonization and invasion of plants (Yoshitake et al., 2013). Larger islands may support more diverse species assemblages of plants than smaller islands because of their greater diversity of habitats and higher habitat quality (Li et al., 2020). Diverse plant communities play irreplaceable functions in shaping microbial communities (Ren et al., 2018). The plant species composition and diversity alter the community composition of microbiota in the soil through the rhizosphere effect (Kowalchuk et al., 2002). Therefore, it is necessary to consider the diversity of plants in shaping microbial communities in volcanic islands. This may be the reason why a higher alpha diversity indices and a more dispersive pattern of beta diversity were detected in Weizhou.

If plant diversity is a directed factor (probably by the rhizosphere effect) in shaping the microbial communities on Weizhou and Xieyang volcanic islands, an indirect factor also must be considered. The higher density and diversity of vegetation on Weizhou Island resulted in more leaf litter. The decomposition of this leaf litter could promote the accumulation of nutrients in the soil. Some scientists have observed that environmental factors, such as temperature and nutrients, determine the diversity or community composition of soil microorganisms (Singh et al., 2012). In this study, although some important physicochemical properties (NO_3_^−^, NH_4_^+^, Fe, TS, TP, TN, and TC) of the soil between Weizhou and Xieyang Island did not vary significantly, we still found a trend in which the concentrations of NO_3_^−^, NH_4_^+^, TN, and TC in Weizhou were higher than those in Xieyang. This differences in soil nutrient levels would affect the composition of the microbial community as well. Our work showed an inclination that NO_3_^−^, NH_4_^+^, Fe, and TP impacted on microbial communities, and NO_3_^−^, NH_4_^+^, TN, and TC exerted a more obvious influence on the actinobacterial populations. However, the influence of nutrients on microbial and actinobacterial communities is not statistically significant. One possible reason is that the number of samples was relatively small (only 12), and perhaps this quantity was not sufficient to illustrate the interaction between environmental factors, such as nutrients, and microbial communities. Another reason is that nutrients could not necessarily explain all of the microbial variation in the soil. Vegetation composition and soil chemistry explain the difference in the variation of the observed microbial population (Mitchell et al., 2012). Some other factors, including but not restricted to the rhizosphere effect, may also significantly shape the microbial communities. However, this impact could not be properly quantified by RDA.

Based on next-generation sequencing (NGS), this study illustrated the actinobacterial populations of volcanic islands in China. The most predominant families of Actinobacteria on the two islands were f_unknown (a group of Actinobacteria could not be identified as any known taxa), Nocardioidaceae, Streptomycetaceae, Pseudonocardiaceae, Micrococcaceae, Propionibacteriaceae, and Frankiaceae. The soil on the two volcanic islands, Weizhou and Xieyang, is primarily laterite, a type of red soil developed from rock-weathering processes (Ge and Han, 2009). Considering the impacts of different habitats on the composition of microbial communities (Ezgi et al., 2021; Borsodi et al., 2022), it is significant to compare our results with the actinobacterial communities in many other soil types. Tang et al. found that the dominant Actinobacteria in the Yanshan Mountains zone of China were Nocardioidaceae, Micromonosporaceae, Conexibacteraceae, Pseudonocardiaceae, Propionibacteriaceae, Acidimicrobineae, Micrococcaceae, Geodermatophilaceae, Streptomycetaceae, Solirubrobacteraceae, Streptosporangiaceae, Mycobacteriaceae, and Acidimicrobiaceae (Tang et al., 2016). In the Badain Jaran Desert and Tengger Desert in China, Geodermatophilaceae, Micrococcaceae, Micromonosporaceae, and Rubrobacteriaceae were the predominant actinobacterial populations (Sun et al., 2018). The actinobacterial community in terrestrial Antarctic microenvironments is dominated by the families Sporichthyaceae, Euzebyaceae, Patulibacteraceae, Nocardioidaceae, and Rubrobacteraceae (Rego et al., 2019). A comparison of the actinobacterial with those in other soil habitats indicated that the actinobacterial community structure in these volcanic islands shared more common characteristics with Actinobacteria in the Yanshan Mountains, where plant-microbe interactions might play a vital role in the development of the ecosystem (Ciccazzo et al., 2014). Volcanic islands are geographically isolated ecosystems. How did Actinobacteria spread from continental mountains to geographically isolated volcanic islands? It is known that the soils of volcanic islands are derived from the weathering of rocks, and the scarcity of microorganisms and nutrient inputs is obvious (Yoshitake et al., 2013; Mora et al., 2014). We are really confused about the formation of microbiota in volcanic island soil. Based on the Actinobacteria analysis, we can hypothesize that pioneer plants, which most likely grew from seeds left by fruit-eating migratory birds, dramatically shaped and altered the soil microbiota of the early volcanic islands (Ciccazzo et al., 2014).

Soil communities may be the most complex environments on Earth. However, more than 95% of the microorganisms in soils are unculturable, and the microbial ecology of soil has generally been treated as a “black box” (Bennett et al., 2019). In the present study, 268 actinobacterial strains were isolated from Weizhou and Xieyang Islands, and they were most related to *Streptomyces* spp., *Micromonospora* spp., *Nocardia* spp., *Amycolatopsis* spp., *Tsukamurella* spp., *Mycobacterium* spp., and *Nonomuraea* spp. The next-generation sequencing (NGS) of 16S rRNA gene amplicons could generate massive amounts of data about environmental microbial communities (Finley et al., 2015; Jesmok et al., 2016). The NGS results indicated that 22.7% of the OTUs belonging to Actinobacteria only shared 88%–95% similarity with the known genera. The unknown taxa restrict our understanding of the functions of Actinobacteria in the soil ecosystem. This subject is worthy of in-depth research to explore novel families, orders, or genera in Actinobacteria. For example, many OTUs formed clades most related to 0319-7 L14 and Nitriliruptoraceae. 0319-7 L14, which was first discovered in Australian arid-zone soil, was designated as an uncultured bacterium (Holmes et al., 2000). Several strains from Nitriliruptoraceae have been isolated from extreme environments, such as hyperhaline (Sorokin et al., 2009) and highly thermophilic (Qiu et al., 2019) habitats. More interestingly, 15 OTUs formed a unique clade and shared identities of less than 95% of their identities with any known families. This is the first report about this unknown group and their 16S rRNA sequences obtained from volcanic soils. These potentially unknown taxa and their appearance in volcanic soils may imply their unique ecological function. Moreover, the “black box” of many unknown Actinobacteria in the Weizhou and Xieyang volcanic soil presents a great challenge and opportunity for us to perform future in-depth studies. Future research should pay more attention to designing novel isolation media for rare community members of Actinobacteria from volcanic islands (Fang et al., 2017). This study provides valuable information for designing media that target specific actinobacterial groups and facilitate the isolation of some Actinobacteria, which were initially challenging to isolate (Sun et al., 2018).

We also found that the actinobacterial groups illustrated by culture-independent methods do not clearly reflect the results obtained by using culture-dependent methods. First, in this study, a total of 141 isolates belonging to 55 species of *Streptomyces* were isolated, representing 73.3% of the total isolated Actinobacteria. However, only 10 OTUs (~3.9%) were classified as *Streptomyces* by HTS analysis. Second, compared to the genus *Tsukamurella* (six isolates) obtained by culture-dependent methods, no OTU was classified as *Tsukamurella* by HTS analysis. This result indicated that there were still knowledge gaps regarding the richness and diversity of Actinobacteria obtained by HTS analysis and their real conditions. Li et al. noted that the DNA extraction and PCR amplification process may result in suppression of the observed proportions of the *Streptomyces* community (Li et al., 2021b). This interpretation is reasonable for explaining the absolutely undetected *Tsukamurella* in volcanic soils using HTS analysis as well. To explore untapped groups of Actinobacteria, such as *Tsukamurella,* a set of universal or genus-specific oligonucleotide primers were necessary for exploring their richness and diversity in the Weizhou and Xieyang volcanic islands (Tan et al., 2006).

In the present research, the bioactivity assay indicated that the actinobacterial strains from Weizhou and Xieyang Island are promising repositories for antibacterial and cytotoxic metabolites. For antibacterial activity, we should pay more attention to the predominant genus *Streptomyces,* especially the most abundant species *S. variabilis* and *S. flavogriseus.* Most of the strains with antibacterial activity were identified as *Streptomyces,* and the maximum proportion were from the predominant species *S. variabilis* and *S. flavogriseus*. This result agrees with our previous finding (Gong et al., 2018), which reminds us that we should put more emphasis on predominant actinobacterial groups during the screening of antibacterial strains. However, this characteristic is not always the case. Mahmoud noted that, in the coral, the most dominant actinobacterial isolates failed to show any antimicrobial activity, whereas less dominant genera, such as *Streptomyces*, did show antimicrobial activity (Mahmoud and Kalendar, 2016). It is widely accepted that antibacterial strains are easier to obtain from *Streptomyces* (Li et al., 2021b). However, increasing evidence indicates that novel antibacterial compounds are increasingly difficult to isolate from *Streptomyces* in conventional environments (Genilloud, 2017). Deep-sea, desert, cryo, and volcanic environments have proven to be unique habitats that are more extreme, and they are excellent sources for discovering novel antibacterial chemicals (Sivalingam et al., 2019). Although the antibacterial activities of *S. variabilis* and *S. flavogriseus* have also been reported by other scientists (Dezfully and Ramanayaka, 2015; Shubha et al., 2017; Wei et al., 2017), those strains were isolated from conventional soil environments. It is still possible that our isolated antibacterial *S. variabilis* and *S. flavogriseus* strains have special physiochemical characteristics and can produce novel metabolites. Moreover, because of the limitation in which only the supernatant of fermentation broth was used for antibacterial screening, we might have missed some strains with antibacterial activity. This consideration may partially explain why relatively fewer antibacterial Actinobacteria were obtained from the volcanic island soil.

Another interesting finding is that many actinobacterial strains with cytotoxic activity were obtained in this work. Moreover, many of them were identified as “rare Actinobacteria,” such as *Tsukamurella*, *Nocardia*, *Nonomuraea*, *Mycobacteroides*, and *Amycolatopsis*. Rare Actinobacteria were defined as taxonomic groups of which many taxa have been greatly underexplored (Namitha et al., 2021; Nofiani et al., 2022). Indeed, many rare Actinobacteria could produce chemically unique metabolites with potent antibacterial or cytotoxic activity and significantly decrease the risk of rediscovery of known chemicals (Ding et al., 2019). To date, the isolation of rare Actinobacteria has mainly focused on extreme and unusual environments (Mohammadipanah and Wink, 2016; Sun et al., 2018) or eukaryotic hosts (Zipperer et al., 2016; Chevrette et al., 2019). Little attention has been given to volcanic islands. This research demonstrated that volcanic islands are an interesting source of “rare Actinobacteria,” and many strains with cytotoxic activity could be extensively discovered from them. In future, on the one hand, new types of media and methods should be designed for the isolation of novel rare Actinobacteria (Fang et al., 2017; Li et al., 2021b; Salam et al., 2021; Xie and Pathom-aree, 2021); on the other hand, genome sequencing and analysis should be conducted to unveil a vast reservoir of biosynthetic gene clusters (BGCs) for natural products in genomes of rare Actinobacteria, which is promising for extensive mining of novel chemicals and unknown biosynthetic mechanisms (van Bergeijk et al., 2020).

## Conclusion

In this study, the diversity of Actinobacteria on the Weizhou and Xieyang volcanic islands was investigated using a high-throughput sequencing and culture-dependent method. In addition, the antibacterial and cytotoxic activities of the Actinobacteria were detected. HTS method was favorable and powerful for illustrating the diversity of Actinobacteria in volcanic island soil. A large unknown and predominant group, most likely a novel family in Actinobacteria, was obtained from volcanic soils. However, the HTS methods did not fully reflect the diversity of Actinobacteria. Interestingly, 15 actinobacterial strains exhibited antibacterial activity against some bacterial indicator strains. The cytotoxicity assay results showed that 27 strains, many of which were identified as rare Actinobacteria, exhibited cytotoxic activity against HeLa and A549 cell lines. This finding illustrated that volcanic islands are vast reservoirs for Actinobacteria with promising antibacterial and cytotoxic activity. Our work provide additional references for the isolation of novel Actinobacteria from some special and untapped environments, or for obtaining some Actinobacteria with antibacterial and cytotoxic properties.

## Data Availability Statement

The datasets presented in this study can be found in online repositories. The names of the repository/repositories and accession number(s) can be found at: https://www.ncbi.nlm.nih.gov/, PRJNA684667.

## Author Contributions

LW and CP: sampling, carried out the experiments, and writing-original draft preparation. ZY, JS, LL, LX, and TY: data curation and carried out the experiments. XW and MY: sampling and carried out the experiments. HX and XL: sampling, writing-reviewing and editing. BG: conceptualization, methodology, writing-original draft preparation, data analysis, and supervision. All authors contributed to the article and approved the submitted version.

## Funding

This research was supported by the National Natural Science Foundation of China (31560727), Guangxi Natural Science Foundation (2018JJA130187, 2020GXNSFDA238015, and 2020GXNSFAA297126), and the funds of The Guangxi Key Laboratory of Beibu Gulf Marine Biodiversity Conservation (2021ZA01).

## Conflict of Interest

The authors declare that the research was conducted in the absence of any commercial or financial relationships that could be construed as a potential conflict of interest.

## Publisher’s Note

All claims expressed in this article are solely those of the authors and do not necessarily represent those of their affiliated organizations, or those of the publisher, the editors and the reviewers. Any product that may be evaluated in this article, or claim that may be made by its manufacturer, is not guaranteed or endorsed by the publisher.

## Footnotes

^1^ http://www.ncbi.nlm.nih.gov

^2^ https://www.ezbiocloud.net/
