# Supplementary material for: Actinobacteria Community and Their Antibacterial and Cytotoxic Activity on the Weizhou and Xieyang Volcanic Islands in the Beibu Gulf of China
Source: Front Microbiol. 2022 Jul 12;13:911408. doi: 10.3389/fmicb.2022.911408 (PMC9317746; doi:10.3389/fmicb.2022.911408)
Supplement: Supplementary file 1 [file Data_Sheet_1.pdf]

Actinobacteria community and their antibacterial and cytotoxic activity on Weizhou and Xieyang volcanic islands in the Beibu Gulf of China

Lin Wang[1]#, Chunyan Peng[1]#, Bin Gong[1]*, Zicong Yang[1], Jingjing Song[1], Lu Li[1], Lili Xu[1], Tao Yue[2], Xiaolin Wang[1], Mengping Yang[1], Huimin Xu[2], Xiong Liu[3]

1. The Guangxi Key Laboratory of Beibu Gulf Marine Biodiversity Conservation, College of Marine Sciences, Beibu Gulf University, Qinzhou, 535011, China

2. Guangxi Key Laboratory of Marine Disaster in the Beibu Gulf, Beibu Gulf University, Qinzhou, 535011, China

3. Sea area use dynamic supervising and managing center of Fangchenggang City, Fangchenggang, 538000, China

# Lin Wang, and Chunyan Peng contribute equally to this article

*Correspondent author:

Bin Gong: gongbin@bbgu.edu.cn

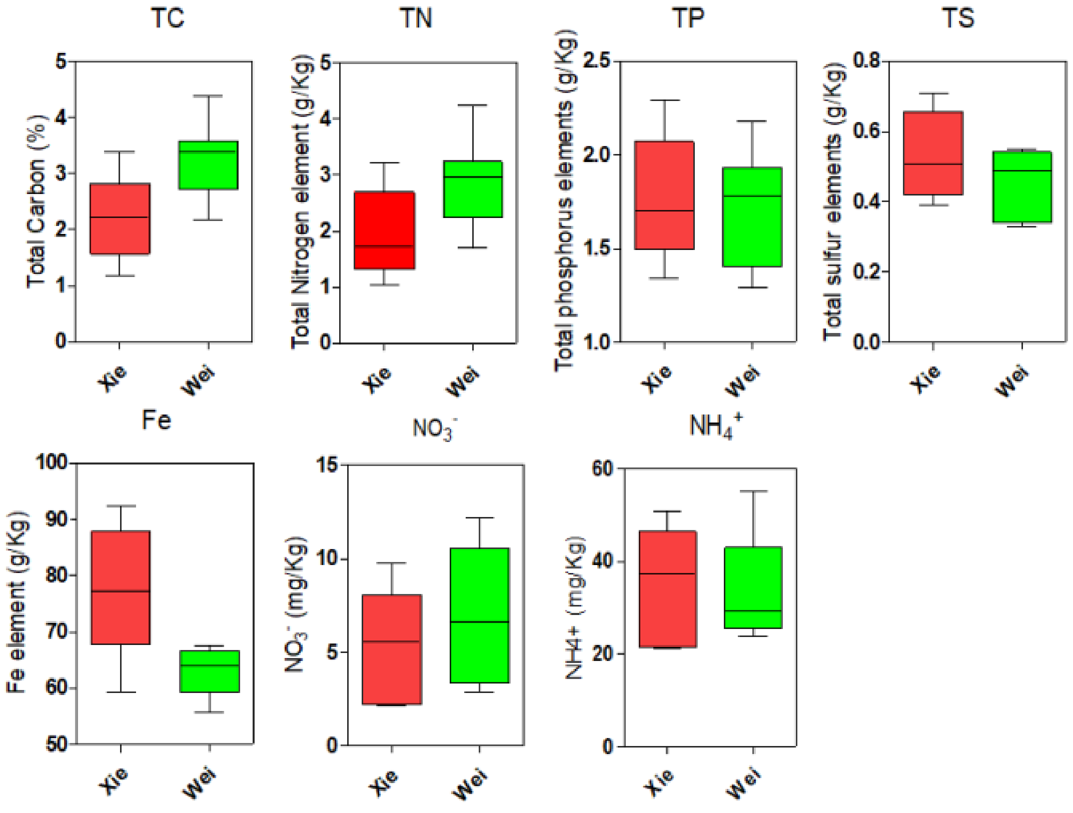

Figure S1. The chemical composition (NO_3^--N, NH_4^+-N, Total carbon contents (TC), Total nitrogen elements (TN), Total sulfur elements (TS), Fe, Total phosphorus elements (TP)) of the soils were measured.
